# A New Approach to Ensuring Oral Health Care for People Living With HIV/AIDS: The Dental Case Manager

**DOI:** 10.5888/pcd9.110297

**Published:** 2012-10-25

**Authors:** Celeste A. Lemay, Suzanne B. Cashman, Anne McDonald, John R. Graves

**Affiliations:** Author Affiliations: Suzanne B. Cashman, University of Massachusetts Medical School, Worcester, Massachusetts; Anne McDonald, Harbor Health Services, Inc, Hyannis, Massachusetts; John R. Graves, Outer Cape Community Health Center, Provincetown, Massachusetts.

## Abstract

**Introduction:**

The American Dental Association has identified several barriers to adequate dental care for vulnerable populations, including appropriate case management. The objective of this study was to examine the perceptions, attitudes, and beliefs of dental patients living with HIV/AIDS on the role and value of the dental case manager (DCM) and the effect of DCM services on their oral or overall health.

**Methods:**

We used a qualitative descriptive study design and focus groups. Twenty-five people who had received DCM services on Cape Cod, Massachusetts, attended 1 of 5 focus groups in 2009 and 2010. Digital recordings of the groups were transcribed verbatim. Textual data were categorized using directed qualitative content analysis techniques. We identified major themes and representative quotes.

**Results:**

The following themes emerged from discussions on the DCM’s role: being available, knowledgeable about clients and insurance, and empathetic; increasing access; and providing comfort. Most participants credited their oral and overall health improvements to the DCM. All participants believed that the DCM was a valuable addition to the clinic and noted that other at-risk populations, including the elderly and developmentally disabled, likely would benefit from working with a DCM.

**Conclusion:**

The addition of a DCM facilitated access to dental care among this sample of people living with HIV/AIDS, providing them with an advocate and resulting in self-reported improvements to oral and overall health.

## Introduction

In 2004, the American Dental Association (ADA) cited several barriers to adequate dental care for vulnerable populations, including the lack of case management services: “[E]ven when [dental] care is available, programs often fail to provide the case management services needed to help people get to dental appointments and comply with post-treatment instructions and oral hygiene protocols” (1).

One vulnerable population for whom access to oral health care is of particular importance is people living with HIV/AIDS (PLWHA). Approximately one-third of PLWHA have difficult-to-treat oral lesions caused by a weakened immune system. Untreated oral disease can affect the ability to eat, thus leading to weight loss and malnutrition (2). For PLWHA, untreated oral disease or inadequate oral health care can be life threatening (3–6).

One approach to improving oral health is use of a dental case manager (DCM). Like the medical case manager (7–9), the DCM functions as a bridge between the patient and care provider. In 2004, the AIDS Institute of the New York State Department of Health suggested using case managers as part of a multidisciplinary approach to increasing access to oral health care for PLWHA (10). DCMs have also been suggested as a way to reduce unmet needs and expand dental services for PLWHA (11).

Several studies have examined the effect of medical case managers in helping vulnerable people to find and remain in care (12–16). Having a medical case manager is associated with a decrease in the number of unmet needs for supportive services, improvement in clinical outcomes (9,10,12–17), and an increase in adherence to medications (16,17). We identified 3 studies on DCMs, 1 focused on a Medicaid population (18) and 2 focused on PLWHA (19,20). In these studies, DCMs helped to increase service use and treatment adherence and provided oral health education. One study (20) concluded — on the basis of an open-ended survey question on how the DCM had been helpful — that PLWHA were satisfied with DCMs’ assistance with access to dental care and provision of comfort. This study did not allow for further examination of DCM activities or their perceived value.

Little has been reported on the experiences of people who have DCMs or their opinions on the effect of DCM services. The objective of this study was to examine the perceptions, attitudes, and beliefs of dental patients living with HIV/AIDS on the role and value of the DCM and the effect of DCM services on their oral or overall health.

## Methods

### Setting

Cape Cod, Massachusetts, classified as rural by the Health Resources and Services Administration, has a diverse population and a well-established gay community. The 2004 Cape Cod Community Survey (21) found that lack of dental care ranked second out of 12 serious household issues. In another survey, PLWHA on Cape Cod identified access to dental care as their second highest need after access to benefits and public assistance (22).

### Dental case managers

We created 2 DCM positions for this study, 1 for each of 2 dental clinics associated with Harbor Health Services, Inc, which received funding from the Health Resources and Services Administration Oral Health Special Projects of National Significance. During the study period, 3 people filled the 2 positions. Two were men; 1 was Brazilian, 1 was white, and 1 was a Hispanic woman. Two were bilingual (English/Spanish and English/Portuguese). Although all had some college education, none had received medical or dental training. They were supervised by a person who had social service experience.

### Design

We used focus groups to collect data. Focus groups are especially helpful in examining nuances in attitudes, beliefs, or opinions (23). We mailed focus group invitations to everyone who had received DCM services at the 2 clinics from November 2007 through November 2009 (n = 216); 28 people agreed to participate, and 25 participated. Participants were required to be aged 18 or older, living with HIV/AIDS, and English-speaking. Using information from prior research (18–20), we drafted a focus group guide; members of a multiprofessional team that included an evaluator, a dentist, and a DCM reviewed the draft questions for appropriateness and comprehension. Questions were revised in response to feedback. Content areas (Appendix) included problems and experiences with obtaining dental care prior to having the DCM; the role of the DCM and ways of improving it; the value of having a DCM; and the effect of the DCM on oral or overall health. We collected self-reported data on sex, race/ethnicity, age, and years since HIV/AIDS diagnosis.

We held 5 focus groups from December 2009 through June 2010. An experienced focus group facilitator used recommended focus group procedures (23) to conduct all 5 groups. The University of Massachusetts Medical School Committee for the Protection of Human Subjects in Research approved study protocols and consent procedures. All participants provided written informed consent and were compensated $25 for participating. Each session was recorded digitally and lasted approximately 1½ hours.

### Analyses

Digital recordings were transcribed verbatim. Transcripts were coded and then imported into Microsoft Excel for sorting and cross-referencing. One investigator read the transcripts several times to identify emerging themes and to develop a coding scheme on the basis of the original research questions and spontaneous comments. Two investigators categorized textual data separately according to directed qualitative content analysis (24).This analysis uses a set of coding procedures for making replicable inferences from data and for identifying emergent themes (25).We calculated the percentage of intercoder agreement and revised the coding scheme after each round until we reached agreement (85%). Disputed responses were reviewed until coders had achieved 100% agreement. Comments expressed most frequently were identified as major themes. We used descriptive statistics to characterize the study sample; compared sex and race/ethnicity of the 25 participants with that of the 216 people eligible to participate, testing differences with the Fisher exact test; and used SPSS Statistics version 19 (IBM Corp, Armonk, New York) for these comparisons.

## Results

### Participant characteristics

Almost all (23/25) participants described themselves as non-Hispanic white; 24 were men; mean age was 49.3 years. Of the population eligible to participate, 86% were male; 85%, non-Hispanic white; 9%, non-Hispanic black (Table 1). We found no significant differences in proportions between participants and the eligible population by sex, white versus nonwhite, or Hispanic versus non-Hispanic. 

**Table 1:** Selected Characteristics of Focus Group Participants and Population Eligible to Participate, Study on Dental Care Management Among People Living With HIV/AIDS, Cape Cod, Massachusetts, 2010^a^

	Focus Group Participants (N = 25)	Population Eligible to Participate (N = 216)
**Sex, n (%)**		
Male	24 (96)	186 (86)
Female	1 (4)	30 (14)
**Race/ethnicity, n (%)**		
Non-Hispanic white	23 (92)	184 (85)
Non-Hispanic black	1 (4)	19 (9)
Other	1 (4)	0
Hispanic	0	13 (6)
**Health insurance, n (%)**		
Public	Data not available	177 (82)
Private		39 (18)
**Age, y**		
Mean (SD)	49.3 (6.5)	Data not available
Range	38–61	Data not available
**Years since HIV/AIDS diagnosis**		
Mean (SD)	11.4 (6.6)	Data not available
Range	2–25	

Abbreviation: SD, standard deviation.

^a ^No significant differences were found in characteristics of 2 populations compared by Fisher exact test: sex (*P* = .22), white vs nonwhite (*P* = .55), and Hispanic vs non-Hispanic (*P* = .37).

### Experiences before receiving DCM services

Participants identified 2 major barriers to obtaining dental care before having a DCM: lack of dental coverage and lack of dental providers. One participant said, “I was always feeling that I was on my own. I didn’t have insurance that covered dental ever, so it was sort of like I was in charge of calling for a cleaning if I had the money” (Participant B, Group 1).

Many participants discussed the need to travel several hours (4 to 6 hours round trip) to get dental care:

Well, there was very little . . . it was still back then, here on Cape Cod, there was very, very little available for Ryan White [Ryan White Dental Treatment Fund pays for dental services for HIV clients who cannot otherwise afford dental care] on the Outer Cape or on the Mid Cape, so you just schlepped, you just went into the city proper, you went into Boston. (Participant A, Group 4)

When help was available, it most often came from medical case managers. That assistance, however, was often limited to making referrals and did not always result in dental care: “And if you have a medical case manager, they can help you get dental care sometimes, if they can find someone to take your insurance if you have MassHealth [Massachusetts Medicaid coverage]” (Participant C, Group 1).

At least 1 participant in every group reported negative experiences with previous dental providers, including receiving poor care, feeling uncomfortable because of HIV status, and receiving untimely care:

I went to [clinic name] in [city name] because you get a discount because you go with a student. The doctors there are teaching the students and it was a horrifying experience. I had my wisdom teeth pulled and there were like 7 people there and they didn’t anaesthetize me and my friend didn’t stay, so they had to just inject me and half of the time it was just wearing out. And the doctor was just the worst training doctor you could probably ever imagine getting and I was just like screaming, you know, and the students were looking and I’m screaming and I had to pull the doctor’s hand away and I was just “What are you doing?” (Participant C, Group 2)

Then I moved up to [city name] with my uncle and up there I couldn’t get any help whatsoever; not for HIV or anything, for absolutely anything, and I got to the point where I pretty much ended up in a nursing home. You know, I was down to 134 pounds. (Participant D, Group 5)

Participants discussed feeling uncomfortable because of their HIV status: “I can’t prove or disprove about the [HIV] status, but in past I have gotten that feeling. You get that, I don’t know how to explain it. You sort of felt, just felt uncomfortable” (Participant B, Group 5); “Well, it becomes important if you feel judged. And I gotta say, that Dr [dentist name], I just don’t feel comfortable with him” (Participant B, Group 4).

### Role of the DCM

Participant descriptions of the DCM role sorted into 6 categories: 1) being accessible and available; 2) being knowledgeable about clients; 3) being knowledgeable about insurance; 4) being empathetic; 5) increasing access to care (ie, scheduling appointments, making appointment reminders, and assuring continuity of care); and 6) providing comfort (Table 2). Participants believed these qualities were not only important to the DCM position but vital to assisting people in obtaining care.

**Table 2:** Positive Characteristics Identified in the Roles of Dental Case Managers (DCMs), Study on Dental Care Management Among People Living with HIV/AIDS, Cape Cod, Massachusetts, 2010

Theme and Exemplary Quotes
**Accessibility and availability**
Their [the DCMs] availability, they’re available, and I can only imagine, if there was something that they should be doing that they’re not doing, something that is not in their area, they could point you in the right direction. (Participant B, Group 5)
I guess availability more than anything, we can call [the DCM] and he answers the phone . . . and so often, you get a receptionist, and they have no clue who you are or what you need, and you have to wait to get an appointment. (Participant C, Group 1)
Yeah, [the DCM] always says call me, if anything is bothering you, give me a call. (Participant B, Group 3)
**Knowledgeable about clients**
I’m just saying that, but yeah, they are much more knowledgeable, more informed than just the regular helping staff. For one thing, AIDS has come to the forefront much more, and we’re getting a lot more specific care and in that regard, certainly I like the fact that they know what they’re talking about. Or that you feel as if they do, and you can talk to them about specifics. (Participant C, Group 5)
Okay, I’m living with HIV, that’s terrifying in [and] of itself. Now I have dental problems that can lead to other conditions, opportunistic infections, you name it, it can happen. And frightening things. You learn that you are more communicable with oral problems, and you’re not aware that maybe you can be sharing things you don’t want to share. So yeah, I will definitely say [they are] knowledgeable. (Participant A, Group 5)
I was taking some [HIV] medicine that gave me a dry mouth, and I told [the DCM], and he coordinated some products for me to have. They are over the counter, but he got me some samples, gave them to me. So I thought that was great. (Participant C, Group 1)
**Knowledgeable about insurance**
Also, I believe that [the DCM] understands under what insurance program you are on, what is available for you, because I was on MassHealth before, and then I had to get off of MassHealth, and so I was directed towards the SPNS program, and he took care of that immediately. There wasn’t any sort of gap, and there was no worry in my mind that I could continue to have care, so it was continued in the same manner, going from one insurance program to another. (Participant D, Group 1)
I think it’s just great that they have the knowledge of all the different programs that are around there that it would just be impossible for a regular person to do research and find them. She was able to find a way, a program that would pick it [cost of upper plate] up. And they made one out of metal instead of acrylic this time. And it feels better, it’s lighter, it’s thinner, I’ve had no problems now. (Participant B, Group 3)
**Empathy**
Oh, yeah, you’re definitely not going in as an outsider. You know that was a big fear. [Dentist name] is my second dentist in my entire life, and I have to believe my former dentist was very conservative, and I dreaded having to go in there with my positive diagnosis, because he had no go with that. I just felt that way. This way there are no problems, there’s not even a hint. (Participant B, Group 2)
He brings up the HIV thing without me . . . because half of the time it’s like okay, now where do I put this in here, do I need to put this in now, or is this the person I’m not supposed to talk to about this? And so he puts that right on the table and wipes that away right away so that you know, this is what we need to know. (Participant C, Group 4)
**Access to care**
I had a problem with one of my teeth, and he was the go-between with the doctor to kind of make sure that I was able to get right in, whereas if you were dealing with receptionists, oh, yeah, well, you know, next week we’ll be able to get in. Whereas he talked to me, and within a half hour he called me back to say, can you come in then? (Participant E, Group 5)
Oh, yeah, I had upper and lower dentures done. It got so bad that they had no choice but to take everything. At Ellen Jones, they wanted to take 2 teeth every month and a half or something like that, which was horrendous, so I just said the heck with that. And then as soon as [the DCM] set me up for an appointment they figured out how to get it all done like within 3 months. (Participant A, Group 2)
Just that little friendly reminder, hi, it’s time to come in again, that’s just a good thing I think. (Participant C, Group 2)
**Providing comfort**
He, I think, set me at ease, you know. I’m afraid of the dentist, I’m afraid of the pain the dentist inflicts sometimes, but I just thought that he, having first gone through that experience he made it comfortable . . . “don’t worry about it, you’re really going to like this.” (Participant B, Group 2)
He, you know, more than anything, it was a comfort feeling, he made me feel comfortable and informed me quite a bit. (Participant D, Group 4)
Are you kidding me, they talk to you. That was the reason I didn’t have dental work done. I had bad teeth as a kid, I didn’t want to go. I was scared to death, you know, or at the very least apprehensive about somebody. They talk you through it. They are so, so cool. Very, very understanding. I hate to say this. You know I’m 50 plus years old, but I was afraid to go to the dentist, okay? (Participant E, Group 5)

A few participants mentioned an additional potential DCM role: assisting people with travel to and from dental appointments:

When I have medical appointments, I can go through the ASG [AIDS Support Group] and get the B bus. But I don’t know if that should be included in the dental [case manager role]. Let’s say, I don’t have much medical, right now it’s more focused on dental, but I still need rides to the [dental clinic] I think. Maybe, to help answer your question of what should be included, maybe that. (Participant C, Group 5)

Only 1 participant mentioned an unmet expectation: “What I’m thinking now is if my dental case manager were to ask me how happy I am with my dental providers, with my dentists” [Participant B, Group 4].

### Value of the DCM

All participants mentioned the value of the DCM; one stated, “I can’t see how a dental practice right now could function without a [dental] case manager” (Participant F, Group 1). Many cited previous difficulties with obtaining dental care, including the complexities of gaining access:

You need someone to centrally coordinate. If you don’t know where to go or what to do, whatever the case may be, then just like you say, you go or you call when you don’t have any idea, so that one person coordinates what is going on or gives direction on what place to go, and this eases the peace of mind first of all. (Participant A, Group 1)

Another participant discussed the value of the position as the coordinator of dental care: “The [dental] case manager is like the overseer, the engineer making sure that not only are we getting the care we need now but we’re also getting the future stuff taken care of” (Participant A, Group 5).

All participants believed that the DCM would benefit other vulnerable populations; one stated:

I can’t imagine how it wouldn’t benefit. I remember when my mother was at the end of her life and when she was in her 70s, especially seniors, where there is so little. I mean, we take such bad care of our elderly. I mean, just in that aspect alone. But I think other populations of the community . . . I can’t imagine how it wouldn’t be helpful. (Participant A, Group 4)

### Effect of DCM on oral health and overall health

Many participants noted changes in their oral and overall health after the DCM helped them to obtain regular dental care. Changes in oral health often involved the alleviation of pain: “Before this I had a half a mouth of teeth for a half a year before I met these dental case managers, so I was suffering, I was slowly losing weight, you know” (Participant D, Group 5). Another participant discussed changes not only in pain reduction but also in laboratory values:

Well, it’s definitely helped me. Because when they did the top teeth, I guess I had an infection in there probably for about a year and a half. So, my T cells once this was all done jumped 100 points. So yeah, and I feel a lot better. Now that I don’t have any pain, I’ve been in pain for like, I don’t know 8 years. I was used to it, you know. (Participant B, Group 2)

Several participants mentioned the effect on their mental health and how poor oral health limited their ability to socialize:

But the whole idea that like when I didn’t have teeth for a while, I couldn’t go to a restaurant, I couldn’t even have a deli sandwich. I’d sit there for 45 minutes, it would take you 20. So, has your health changed? Health, mental health, physical health, you know — everything. (Participant E, Group 5)

Overall, participants felt that their improved oral health had improved their overall health.

## Discussion

The issues and problems of oral health access before receiving DCM services that our study’s participants described are consistent with previous findings (22) and highlight the persistence of barriers to oral health care for vulnerable populations on Cape Cod. Although participants said they had medical case managers, they said these managers did not facilitate access to oral health care. The DCM was mentioned in all groups as having a role in increasing access to oral health care.

Although modeled after medical case managers, DCMs, as described by our study participants, may be more like community health workers (CHWs). The American Public Health Association CHW Special Interest Group defines a CHW as

. . . a frontline public health worker who is a trusted member of and/or has an unusually close understanding of the community served. This trusting relationship enables the CHW to serve as a liaison/link/intermediary between health/social services and the community to facilitate access to services and improve the quality and cultural competence of service delivery. (26).

The role of the DCM, as described by our study participants, incorporates many of these responsibilities, including being available, empathetic, and knowledgeable about their clients’ needs and providing comfort. The DCMs in our study did not have degrees in medicine, dentistry, or other health care fields; they were community members. CHWs have been effective in increasing access to medical care, particularly for vulnerable populations (27–29); a similar position in dental clinics may benefit all people who need access, not just PLWHA.

The Surgeon General in 2010 described the direct connection between oral health and general health, acknowledging the relationship between chronic oral infections and chronic diseases such as HIV/AIDS, diabetes, osteoporosis, and cardiovascular conditions (30). Many of our study participants described not only improvements in oral health after access to a DCM but also improvements in overall physical and mental health. Our findings suggest the need to incorporate DCMs into dental clinics and integrate oral health into overall health care and into public health infrastructure.

This study has several limitations. First, because we used focus groups, which allowed us to obtain in-depth answers to open-ended questions, we had a small sample. Second, our results may reflect participation bias; the study participants may not accurately represent all dental clients receiving DCM services. Third, a study conducted in only 1 place limits generalizability. The generalizability of our data is especially limited because we collected data on only a few demographic characteristics, and these characteristics did not reflect the demographics of the overall population of interest (ie, Latino and black men, who outnumber white men in new HIV/AIDS cases [31]). Fourth, we did not explore the question of cost-effectiveness, an area for future research. Quantitative measures, such as the brief Oral Quality of Life Measure (32), would also be valuable in future studies of DCMs.

The addition of a DCM to 2 dental clinics on Cape Cod, Massachusetts, facilitated access to dental care and provided PLWHA with a health care advocate, resulting in self-reported improvements to oral and overall health. Participants believed that the DCM was a valuable addition to the clinic and noted that other at-risk populations (eg, people with diabetes, the elderly, the developmentally disabled) would likely benefit from working with a DCM.

## Appendix. Focus Group Interview Guide Key Questions

A. In the past, prior to having a dental case manager, have you experienced any problems or difficulties in obtaining the dental care you needed? If things went well, could you tell us in what ways they ran smoothly? If things did not go well, in what ways did they not run smoothly?

Did anyone help you receive the care you needed?
How did they help?

B. What do you think the role of the dental case manager should be?

What type of activities do you think the dental case manager should carry out?
In what ways does your DCM meet (or not meet) these expectations? Please explain.

C. Has your dental case manager helped you receive the dental care you needed?

If yes, how have they helped?
If no, what could they have done to help you receive the care you needed?

D. Has your dental case manager helped you in any other ways?

Can you provide an example of how your dental case manager has helped you?

E. How has your oral health changed since you have had a dental case manager available to you?

Have you experienced any changes in your overall health since you have been receiving assistance from a dental case manager (includes physical health, emotional health)?

F. Were there ever times that you found yourself wishing the dental case manager would do something or would help you in some way when he/she did not? Could you elaborate and explain, please?

G. Finally, using a dental case manager to help people receive the dental care they need is not a traditional way of reaching out to people. Do you think that dental case managers are a valuable addition to the dental clinic? Why or why not?

H. Does anyone have any additional comments they would like to share?
